# Viral RNA Intermediates as Targets for Detection and Discovery of Novel and Emerging Mosquito-Borne Viruses

**DOI:** 10.1371/journal.pntd.0003629

**Published:** 2015-03-23

**Authors:** Caitlin A. O’Brien, Jody Hobson-Peters, Alice Wei Yee Yam, Agathe M. G. Colmant, Breeanna J. McLean, Natalie A. Prow, Daniel Watterson, Sonja Hall-Mendelin, David Warrilow, Mah-Lee Ng, Alexander A. Khromykh, Roy A. Hall

**Affiliations:** 1 Australian Infectious Disease Research Centre, School of Chemical and Molecular Biosciences, The University of Queensland, St. Lucia, Queensland, Australia; 2 Public Health Virology Laboratory, Forensic and Scientific Services, Department of Health, Archerfield, Queensland, Australia; 3 Department of Microbiology, National University Health System, National University of Singapore, Singapore; George Mason University, UNITED STATES

## Abstract

Mosquito-borne viruses encompass a range of virus families, comprising a number of significant human pathogens (e.g., dengue viruses, West Nile virus, Chikungunya virus). Virulent strains of these viruses are continually evolving and expanding their geographic range, thus rapid and sensitive screening assays are required to detect emerging viruses and monitor their prevalence and spread in mosquito populations. Double-stranded RNA (dsRNA) is produced during the replication of many of these viruses as either an intermediate in RNA replication (e.g., flaviviruses, togaviruses) or the double-stranded RNA genome (e.g., reoviruses). Detection and discovery of novel viruses from field and clinical samples usually relies on recognition of antigens or nucleotide sequences conserved within a virus genus or family. However, due to the wide antigenic and genetic variation within and between viral families, many novel or divergent species can be overlooked by these approaches. We have developed two monoclonal antibodies (mAbs) which show co-localised staining with proteins involved in viral RNA replication in immunofluorescence assay (IFA), suggesting specific reactivity to viral dsRNA. By assessing binding against a panel of synthetic dsRNA molecules, we have shown that these mAbs recognise dsRNA greater than 30 base pairs in length in a sequence-independent manner. IFA and enzyme-linked immunosorbent assay (ELISA) were employed to demonstrate detection of a panel of RNA viruses from several families, in a range of cell types. These mAbs, termed monoclonal antibodies to viral RNA intermediates in cells (MAVRIC), have now been incorporated into a high-throughput, economical ELISA-based screening system for the detection and discovery of viruses from mosquito populations. Our results have demonstrated that this simple system enables the efficient detection and isolation of a range of known and novel viruses in cells inoculated with field-caught mosquito samples, and represents a rapid, sequence-independent, and cost-effective approach to virus discovery.

## Author Summary

This paper describes a simple and cost-effective system for screening biological samples for virus-infection. The authors demonstrate the application of two antibodies to detect double-stranded RNA (dsRNA) which is a common molecule produced in infection by a number of different viruses. The use of antibodies which react with double-stranded RNA independently of sequence allows for detection of a diverse range of viruses and has been instrumental in the detection of known arboviruses from three different families and the discovery of a number of previously unknown viruses from Australian mosquito populations. This system provides a rapid and economical approach to virus surveillance and discovery. This is the first report of anti-dsRNA antibodies used in a streamlined system for virus detection and discovery in field-caught samples.

## Introduction

Arthropod-borne viruses (arboviruses) encompass a range of veterinary and medically significant viral pathogens belonging to five antigenically distinct families of RNA viruses. These families can be separated according to their genome type: those with positive-sense single-stranded RNA ((+)ssRNA) genomes, the *Togaviridae*, *Flaviviridae*; negative-sense RNA ((-) ssRNA) genomes, *Rhabdoviridae* and *Bunyaviridae*; and the double-stranded RNA (dsRNA) viruses of the *Reoviridae* family.

These viruses cycle between haematophagous arthropod vectors and reservoir/amplifying vertebrate hosts. Occasionally humans and livestock can become incidental hosts for these viruses and may develop encephalitic or haemorrhagic disease. New and more virulent strains of these viruses are continually emerging and expanding their geographic range [1, 2]. As a result many arthropod populations are routinely surveyed in an attempt to assess the risk of arboviruses and identify emerging pathogens.

The co-circulation of insect-specific viruses, such as the divergent insect-specific flaviviruses (ISFs), adds another layer of complexity to the spread and distribution of arboviruses in mosquito populations [3]. While not of direct affect to the health of humans and animals, our lab and others have shown that ISFs circulating in mosquito populations may suppress or enhance the replication of pathogenic arboviruses such as the encephalitogenic West Nile virus (WNV) [4–6].

Surveillance for arboviruses and detection of new mosquito-borne viruses currently relies on antigenic, molecular or deep sequencing based approaches [7–11]. However, these methods are often expensive or limited by genus-specificity and divergent viruses such as ISFs are often missed. We have developed a novel assay system based on two unique monoclonal antibodies (mAbs) that recognise an antigen in cells infected with a wide range of viruses. This system provides a streamlined and economical approach for virus detection and discovery. Here we characterise the antigen recognised by these novel mAbs and show that this system provides a streamlined method for detecting infection with viruses from at least three of five conventional arboviral families as well as a new species in the novel family *Mesoniviridae*.

## Materials and Methods

### Production and characterisation of monoclonal antibodies

Hybridomas secreting mAbs 3G1 and 2G4 were generated by immunising an adult female BALB/c mouse with concentrated supernatant from C6/36 cell cultures infected with Palm Creek virus as previously described [6]. Antibodies were isotyped using Mouse Typer Isotyping kit (BioRad) as per the manufacturer’s instructions and then confirmed by electron microscopy. Monoclonal antibodies 2G4 and isotype control 3.1112G [12] were purified using GE IgM purification HP column (1 ml) (17–5110–01) as per manufacturer’s instructions with 1 M ammonium sulfate using AKTA FPLC system. Antibody 3G1 was unable to be purified by this method. In order to obtain a crudely purified stock of this mAb, 3G1 hybridoma cell culture supernatant was concentrated using a high molecular weight (300K) MWCO Spin-X UF concentrator column (Corning).

### Electron microscopy

Preparations of purified 2G4 and concentrated 3G1 were diluted to a final concentration of 5 μg/ml in PBS. 4 μl samples of the antibody preparations was pipetted onto glow-discharged carbon-formvar films supported on 400-mesh copper-grids and negative staining was performed using 1% uranyl acetate. Samples were imaged at 59000x magnification using a Tecnai F30 FEG-TEM (FEI) operating at 300 kV with a 4k lens-coupled camera (Direct Electron).

### Cell and virus culture

C6/36 (*Aedes albopictus*) cells [13, 14] were maintained in RPMI-1640 supplemented with 5% fetal bovine serum (FBS) and grown at 28°C. Vero cells (African Green Monkey Kidney) [15] were maintained in Dulbecco’s modified Eagle’s medium (DMEM) containing 2–5% FBS, while DF-1 cells (chicken embryo fibroblast) [16] were grown in DMEM with 5% FBS. Hybridoma cell lines used in this study were grown in hybridoma serum free medium (Invitrogen) initially supplemented with 20% FBS and then weaned to serum-free culture for antibody production. All vertebrate cells were incubated at 37°C with 5% CO_2_. All media were supplemented with 50 μg/ml streptomycin, 50 U/mL penicillin and 2 mM L-glutamine.

The viruses used in this study were West Nile virus subtype Kunjin strain MRM61C (WNV_KUNV_), West Nile virus strain New York 99 (WNV_NY99_), dengue virus serotype 1 (DENV-1), dengue virus serotype 2 strain New Guinea C (DENV-2 NGC), Yellow Fever virus strain 17D (YFV 17D), Murray Valley encephalitis virus strain 151 (MVE 151), Palm Creek virus 56 (PCV), Ross River virus T48 (RRV), Barmah Forrest virus (BFV), Akabane virus strain A661 (AKAV; obtained from Peter Kirkland, EMAI), bovine ephemeral fever virus strain F704 (BEFV; from Peter Kirkland, EMAI), Blue Tongue virus isolate D870 (BTV; obtained from Peter Kirkland, EMAI).

All virus stocks were prepared from the cell culture supernatant of virus-infected *Aedes albopictus* (C6/36) or baby hamster kidney (BHK) for those that did not grow in C6/36 cells (AKAV, BEFV, BTV) as per previously described protocol [17]. The virus titre was determined as 50% tissue culture infective dose (TCID_50_) by fixed-cell ELISA using virus specific mAbs [17].

### Heat shock treatment

C6/36 cells were seeded in 96-well plates and infected the following day with WNV_KUNV_ at a multiplicity of infection (MOI) of 1 or mock infected. At day 8 post seeding, a subset of mock infected cells were incubated at 41°C for 3 hours to induce heat shock. Immediately post heat shock treatment all cells were fixed with acetone fixative buffer (20% acetone with 0.02% bovine serum albumin (BSA) in phosphate buffered saline (PBS)). Control mock-infected and WNV-infected cells were maintained at 28°C for the duration of the study.

### Fixed cell ELISA using mAbs 3G1 and 2G4

Fixed-cell ELISA was performed as per previously published methods with slight modifications [18]. Briefly, infected or mock-infected cell monolayers were fixed with acetone fixative buffer. Plates were blocked with 150 μl per well ELISA blocking buffer (0.05 M Tris/HCl (pH 8.0), 1 mM EDTA, 0.15 M NaCl, 0.05% (v/v) Tween 20, 0.2% w/v casein) for 1 hour at room temperature before probing with 50 μl/well respective mAbs diluted in blocking buffer as needed. Plates were incubated with primary antibody for 1 hour at 37°C before washing 4x with PBS containing 0.05% tween-20 (PBST). Secondary antibody goat anti-mouse HRP (DAKO) was diluted 1/2000 in blocking buffer and added at 50 μl/well. After another incubation at 37°C for 1 hour, plates were washed 6x with PBST. Finally, 100 μl/well substrate solution [1 mM 2,2-azino-bis(3-ethylbenzthiazoline-6-sulfonic acid) (ABTS), 3 mM H_2_O_2_ in a buffer prepared by mixing 0.1 M citric acid with 0.2 M Na_2_HPO_4_ to give a pH of 4.2] was added per well and plates were incubated in the dark at room temperature for 1 hour. Absorbance was measured at 405 nm.

### Immunofluorescence assays (IFA)

All cells were grown on glass coverslips, mammalian cells were seeded at 98% confluency one day prior to infection. C6/36 cells were seeded at 25% confluency to allow for longer incubation times. Monolayers were infected or transfected as indicated and fixed with ice cold 100% acetone for 5 minutes and air dried before storing at -20°C. Prior to staining, coverslips were blocked with 1% BSA/PBS for one hour at room temperature. For single antibody staining, coverslips were incubated for 1 hour at 37°C with 3G1 and 2G4 hybridoma cell culture fluid or virus specific mAbs as follows: mAb 3.1112G, anti-non-structural (NS) protein 1 (reactive with WNV, IgM) hybridoma culture fluid; pan-flavivirus mAb 4G2 anti-envelope (E) (IgG) [19] hybridoma culture fluid; mAb r847 anti-BTV hybridoma culture fluid (obtained from Peter Kirkland, EMAI); mAb J2, anti-dsRNA used at 1:200 (IgG, English and Scientific Consulting, Hungary) [20]. Coverslips were washed 3x with PBS and stained with Alexafluor 488-conjugated goat anti-mouse IgG (H+L) (Invitrogen) diluted 1:500 in 1%BSA/PBS and Hoechst 33342 nuclear stain (Invitrogen) for 1 hour or 5 minutes at room temperature respectively. Following another 3 washes with PBS, the coverslips were mounted onto glass microscope slides using ProLong Gold Anti-fade (Invitrogen). For dual staining, coverslips were first incubated for 1hr RT with virus specific antibodies as follows: anti-non-structural (NS) protein 3 [21] (diluted 1:100, polyclonal rabbit sera, flavivirus-reactive); mAb 5H1 anti-NS5 antibody hybridoma cell culture supernatant [22] (IgG, strong reaction with WNV_KUN_ strains, weak for WNV_NY99_, non-reactive with WNV strains from other lineages); mAb 4G4 anti-NS1 [18] (IgG, pan-flavivirus); mAb G8 anti-RRV E2 antibody hybridoma cell culture supernatant [23]; mAb r5412 anti-AKAV hybridoma cell culture supernatant (obtained from Peter Kirkland, EMAI); mAb r1459 anti-BEFV hybridoma cell culture supernatant (obtained from Peter Kirkland, EMAI). Coverslips were then washed 3x with PBS and stained with Alexafluor 488-conjugated goat anti-mouse IgG (H+L), or 3x with PBST and stained with Texas Red-conjugated goat anti-rabbit IgG (H+L) (Invitrogen) for anti-NS3 sera for 1 hour RT. Coverslips were again washed 3x with PBS and incubated with 3G1 or 2G4 hybridoma culture supernatant for 1 hour 37°C. Finally, coverslips were washed 3x with PBS and stained with 594-conjugated goat anti-mouse IgM (μ chain) or 488-conjugated goat anti-mouse IgM (μ chain) (for monolayers stained with rabbit sera) (Invitrogen) for 1 hour at 37°C followed by Hoechst 33342 nuclear stain (Invitrogen) for 5 minutes before washing and mounting with ProLong Gold Anti-fade. All coverslips were viewed under the ZEISS LSM 510 META confocal microscope.

### RNA extraction and PCR analyses

For isolation of viral RNA from supernatant of inoculated cells, RNA extraction was performed using Machery-Nagel viral RNA isolation kit, while Tri Reagent (Sigma) was used to harvest lysates of virus-infected C6/36 cells in 24 well plates and RNA was extracted according to the manufacturer’s instructions. RT-PCR for identification and confirmation of known viruses was performed using virus-specific primers (Flavivirus (FU2: GCTGATGACACCGCCGGCTGGGACAC, cFD3: AGCATGTCTTCCGTGGTCATCCA); Alphavirus (F1: TTTAAGTTTGGTGCGATGATGAAGTC, R: GCATCTATGATATTGACTTCCATGTT); Sindbis virus (SINVF7: GCCAGAGTGTGTCTCAAGCA, SINVR7: TCGATTCGTTCCTTCCACTT)) in the SuperScript III One-Step RT-PCR System with Platinum Taq DNA polymerase (Invitrogen) [24–26]. Quantitative RT-PCR to assess RNA extracted from DENV-2 and WNV_KUNV_-infected C6/36 lysates was performed using Taqman RT-PCR assays as described by Warrilow et al (2002) and van den Hurk et al (2014) [27, 28].

### Screening of mosquito homogenates

Screening was performed by inoculating filtered mosquito homogenate onto monolayers of C6/36 cells in 96-well microplates and incubating at 28°C for 5–7 days. Cells were monitored over 7 days for cytopathic effect (CPE). At 7 days post-infection culture supernatant (200 μL) was collected and stored at −80°C for further analysis and cells were then fixed using acetone fixative buffer. ELISA was then performed on fixed cells as specified previously using a cocktail of mAbs 3G1 and 2G4.

### Nucleic acid ELISAs

Anti-poly(I:C) ELISA was performed as per Schonborn et al (1991) [20]. Briefly, a 96-well plate was coated with 150 μl/well 2% w/v protamine sulfate (Sigma-Aldrich) in PBS and incubated at 37°C for 2 hours. Plates were then incubated with 100 μl/well (0.25–200 ng) Poly(I:C) diluted in TE Buffer (100 mM Tris, 1 mM EDTA, pH 8) for a further 2 hours at 37°C. ELISA was performed as previously described using 3G1 and 2G4 hybridoma culture fluid and purified anti-dsRNA antibody J2 (diluted at 1:500 in ELISA blocking buffer, English and Scientific Consulting, Hungary).

Capture ELISA was performed by coating purified mAbs at a pre-determined optimal range (0.5–1 μg) in PBS and incubating overnight 4°C. Small biotinylated dsRNAs (30, 40 and 50 bp designed to KUNV NS1, IDT) were diluted (0.25–50 pmol) in TE buffer, added 50 μl/well and incubated 2 hours 37°C. Streptavidin conjugated with horse radish peroxidase (HRP) was diluted 1/2000 in PBST and added 50 μl/well.

### Poly(I:C) transfections

Cells were seeded to 98% confluency on coverslips in 24-well plates. The following day, cells were washed 3x with sterile PBS and topped up with media without Pen/Strep. 2 μl lipofectamine 2000 (Invitrogen) and 2.5 μg Poly(I:C) were added to each well with OptiMEM media. Transfections were incubated for 6 hours and then fixed with ice cold 100% acetone.

### RNAse III digestion

Shortcut RNAse III (New England BioLabs) digestion was performed as per manufacturer’s suggestion. Briefly, 150 μl enzyme preparation (2U/100 μl) or buffer without enzyme (mock digestion) was added to acetone-fixed cell monolayers on coverslips and incubated at 37°C for 2 hours before removing and staining for IFA as above.

### Sensitivity assays for MAVRIC ELISA

C6/36 cells at 80–90% confluency were infected with WNV_KUNV_, RRV or mock-infected using serial dilutions of virus (10^-1^ to 10^-12^). Infected cells were fixed at 24 and 48 hours post infection and 5 days post-infection using acetone fixative buffer. Fixed cell ELISA was performed using mAbs 3G1 and 2G4 and pan-flavivirus anti-E protein mAb 4G2 or mAb G8 (anti-RRV E2). ELISA using each antibody was performed on triplicate plates and the titre of virus detected by each antibody was determined using the Reed-Meunch calculation.

## Results

### Novel monoclonal antibodies recognise an antigen in cells infected with a diverse range of viruses from different viral families

**Fig 1:**
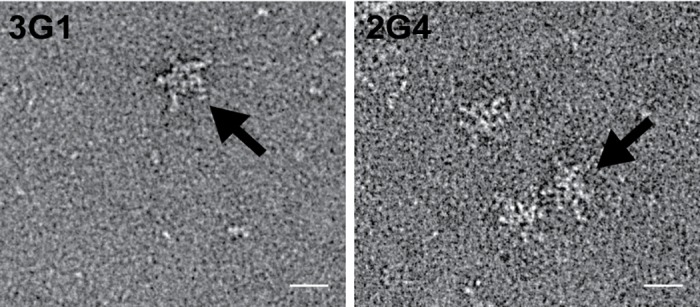
Negative staining electron micrograph of mAbs 3G1 and 2G4. Antibody preparations were diluted in PBS and negatively stained with 1% uranyl acetate before imaging at 59000x magnification. Scale bar depicts 20 nm. Arrows denote a typical IgM pentamer confirmation.

Monoclonal antibodies (mAbs) 3G1 and 2G4 were raised while generating a panel of antibodies to the ISF Palm Creek virus (PCV) [6]. Electron microscopy analysis revealed that the antibodies formed cyclic complexes approximately 30 nm in diameter with five radially protruding arms consistent with the features of pentameric IgM antibodies (Fig. 1) [29, 30]. Our analysis showed that these antibodies reacted strongly with PCV-infected *Aedes albopictus* (C6/36) cells in fixed-cell ELISA, as well as against C6/36 cells infected with a number of pathogenic flaviviruses compared to mock-infected cells (Table 1). Further analysis showed mAbs 3G1 and 2G4 also strongly reacted in fixed-cell ELISA against C6/36 cells infected with the alphaviruses Ross River virus (RRV) and Barmah Forrest virus (BFV) (Table 1). The ability of these antibodies to detect infection with viruses from two antigenically distinct families suggested that the antibody target was unlikely to be of specific viral origin and may be a factor produced as the result of infection with RNA viruses.

**Table 1:** Reactivity of mAbs 3G1 and 2G4 against acetone-fixed C6/36 cells infected with a panel of flaviviruses and alphaviruses in ELISA or IFA.

Virus tested	3G1	2G4
PCV56	+	+
WNV_KUNV_	+	+
WNV_NY99_	+	+
MVE-151	+	+
YFV 17D	+	+
DENV-1	+*	+*
DENV-2	+*	+*
RRV	+	+
BFV	+	+

Legend: +, positive;-, negative;

*, IFA result.

PCV56, Palm Creek Virus prototype; WNV_KUNV_, West Nile virus Kunjin subtype; WNV_NY99_, West Nile virus New York 99 strain; MVE, Murray Valley encephalitis virus; YFV, Yellow Fever virus; DENV-1, dengue virus serotype 1; DENV-2, dengue virus serotype 2 strain NGC; RRV, Ross River virus; BFV, Barmah forest virus.

### 3G1 and 2G4 binding is not increased in heat-shocked cells

**Fig 2:**
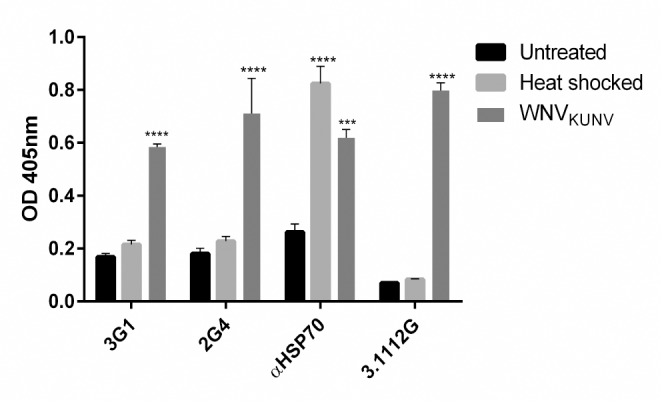
Reactivity of mAbs 3G1 and 2G4 to heat shocked cells. Fixed-cell ELISA was performed on C6/36 cells infected with WNV_KUNV_ MOI: 1 7days post-infection, mock-infected or heat shocked by incubating at 41°C for 3 hours. Monoclonal antibodies used were 3G1 and 2G4, WNV_KUNV_ NS1-specific mAb 3.1112G (IgM) or insect anti-heat shock protein 70 (HSP70) mAb. **** denotes significant difference between test samples and the negative control (mock). Statistical significance was analysed using two-way ANOVA, followed by Dunnett test for multiple comparisons (P<0.0001).

In order to assess whether mAbs 3G1 and 2G4 were recognising a general stress-response protein, their reactivity against heat-shocked C6/36 cells was assessed. In fixed-cell ELISA, mAbs 3G1 and 2G4 reacted strongly against WNV_KUNV_-infected cells, but did not show a significant increase in reactivity against heat-shocked cells (Fig. 2). Anti-heat shock protein 70 (HSP70) mAb showed significant reactivity against both heat-shocked and virus-infected cells (Fig. 2), indicating that HSP70 up-regulation was induced in these cells by both heat-shock treatment and viral infection as previously shown [31, 32].

### 3G1 and 2G4 detect viral infection in vertebrate cell lines

**Fig 3:**
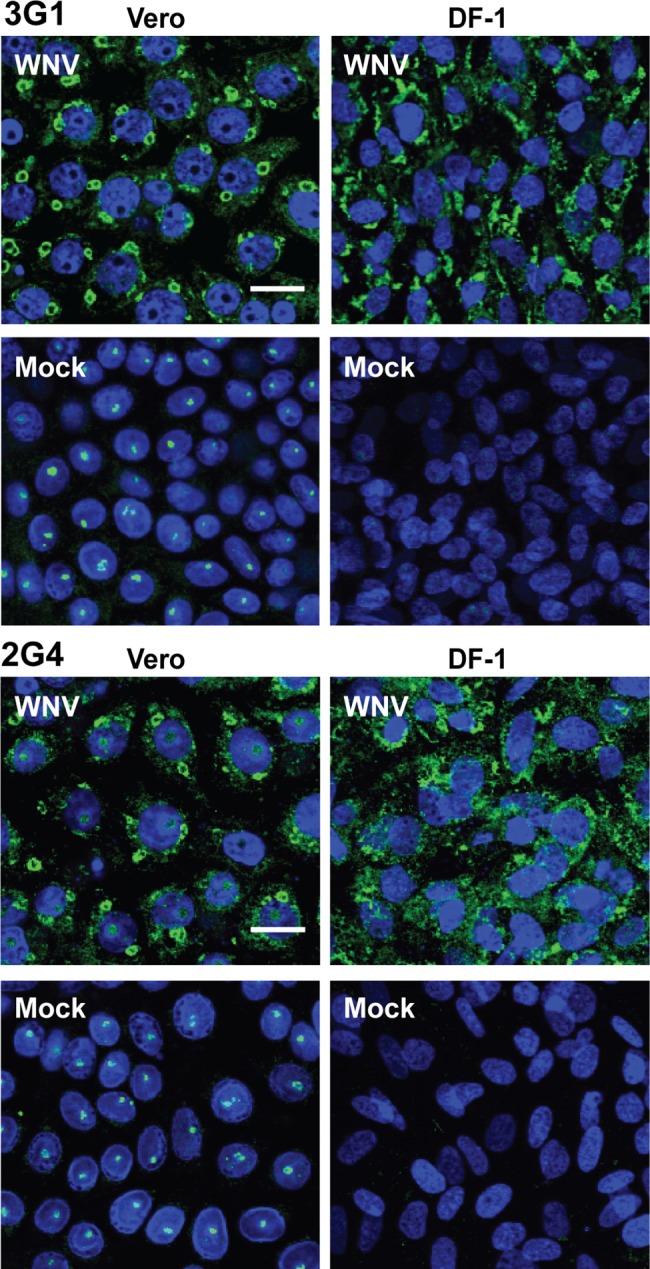
3G1 and 2G4 reactivity to flavivirus-infected vertebrate cell lines. Immunofluorescence assay performed on vertebrate cell lines African Green monkey cells (Vero) and chick embryo fibroblasts (DF-1) mock-infected or WNV_KUNV_-infected MOI: 10 and fixed at 48 hours post infection. MAbs 3G1, 2G4 and 3.1112G were labelled with goat anti-mouse Alexafluor 488 (green). Nuclei are labelled with Hoechst nuclear stain (blue). Slides were imaged at 40x magnification. Scale bar denotes 10 μm.

Given the wide range of viruses detected by 3G1 and 2G4 in mosquito cells and the ability of these viruses to infect other cell types, we decided to assess whether these mAbs would react against WNV_KUNV_-infected mammalian (Vero) and avian (DF-1) cells in IFA (Fig. 3). Staining in infected DF-1 and Vero cells by both mAbs appeared in the perinuclear region. At 48 hours post infection with WNV_KUNV_, strong punctate circles were obvious in the perinuclear region of Vero cells stained with 3G1 and 2G4 (Fig. 3). Similar patterns were also observed in cells stained with isotype control antibody 3.1112G which is specific to WNV non-structural protein 1 (anti-NS1). This circular perinuclear staining observed for both 3G1 and 2G4 in WNV_KUNV_-infected Veros is consistent with staining for flavivirus RNA replication complexes formed inside vesicle packets in the endoplasmic reticulum of infected cells [21, 33], suggesting that the antigen was associated with viral RNA replication.

Both antibodies exhibited some nuclear staining in uninfected Vero cells, which appeared to be associated with the nucleolus (Fig. 3).

### Antigen recognised by 3G1 and 2G4 is associated with flavivirus replication

**Fig 4:**
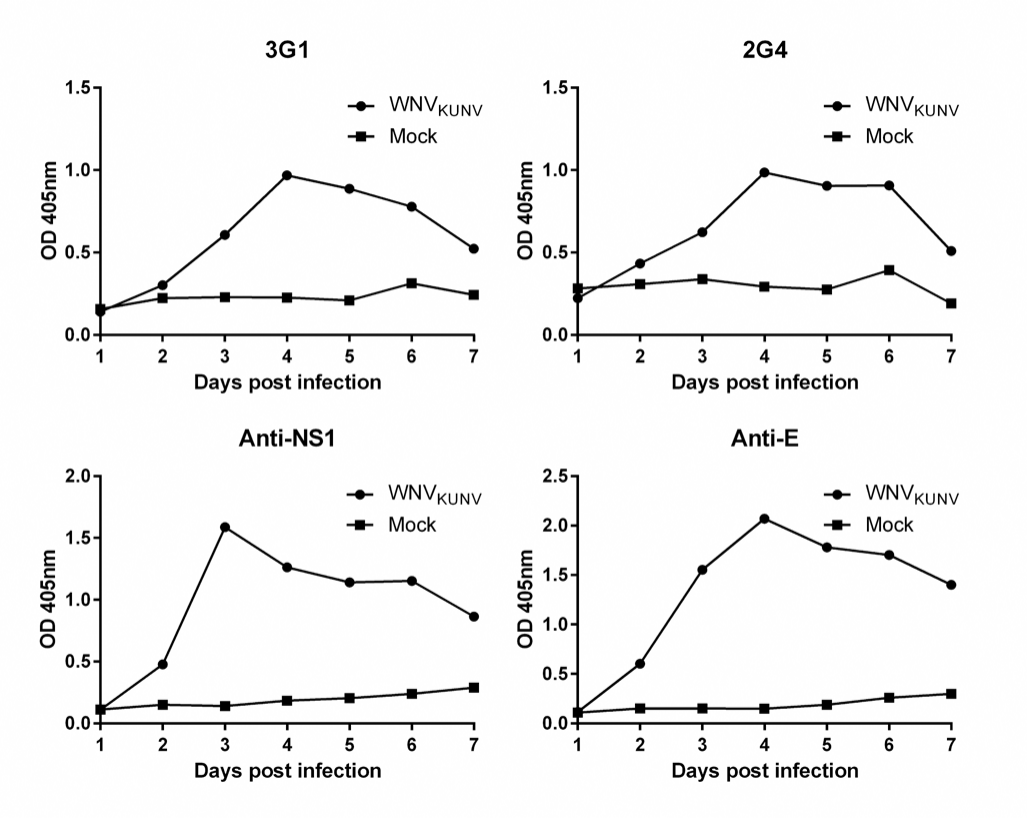
Time kinetics of mAb-reactive antigen in flavivirus infected cells. Fixed-cell ELISA was performed on C6/36 cells infected with WNV_KUNV_ at MOI: 0.01 or mock-infected fixed over 7 days. Antibodies used were mAbs 3G1 and 2G4, flavivirus NS1-specific mAb 4G4 or flavivirus E protein-specific mAb 4G2.

To further investigate the involvement of the mAb-reactive antigen with flavivirus replication, an antibody-binding assay was performed to look at the timing of its induction during infection. This assay revealed that binding of both mAbs 3G1 and 2G4 to WNV_KUNV_-infected C6/36 cells peaked at 4 days post-infection (Fig. 4). This binding pattern mirrored the detection of viral NS1 protein which peaked at 3 days post-infection and envelope (E) protein which peaked at 4 days post-infection (Fig. 4).

**Fig 5:**
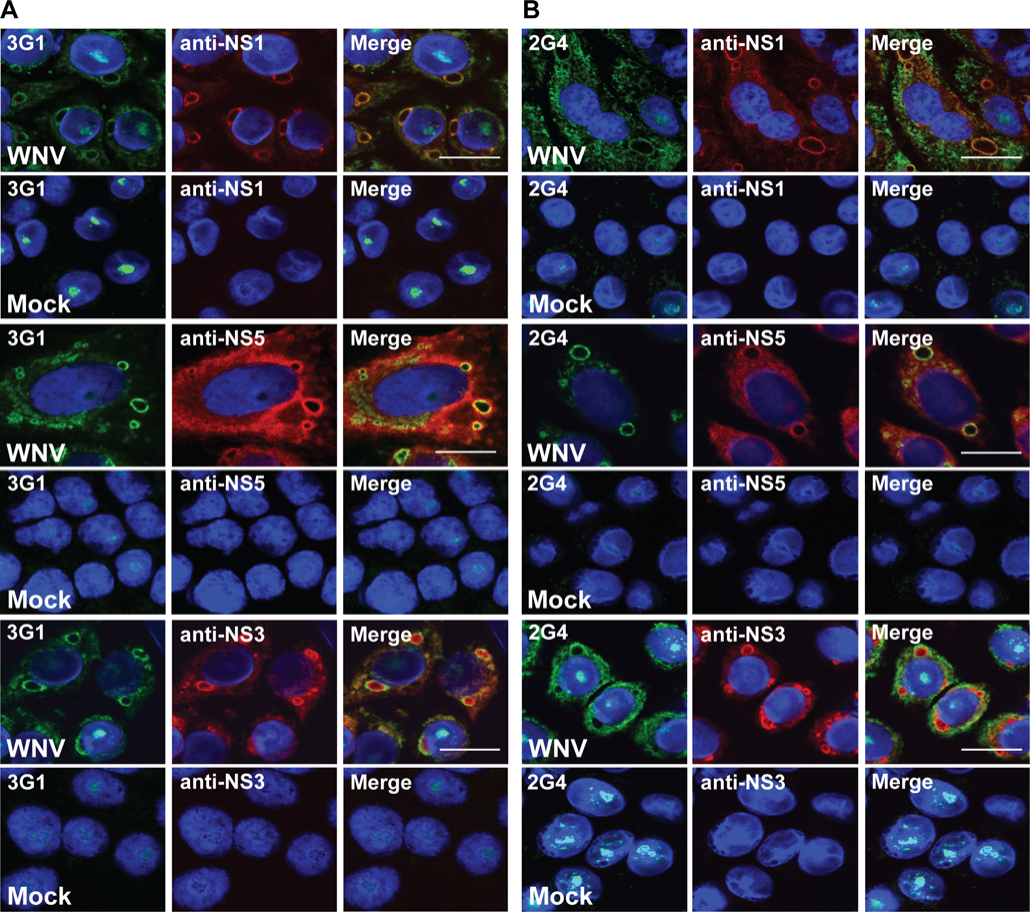
Co-localisation of mAb 3G1 and 2G4 staining with flaviviral replication complexes. Dual was staining performed on Vero cells infected with WNV_KUNV_ MOI: 10 or mock-infected and acetone-fixed at 48 hours post-infection. Merged images showing co-localisation of A) 3G1 (green), and B) 2G4 (green) with flavivirus NS1 labelled with anti-NS1 mAb (4G4, red), flavivirus NS5 labelled with anti-NS5 mAb (5H1, red) and flavivirus NS3 labelled with polyclonal anti-NS3 rabbit sera (red). Nuclei stained with Hoechst nuclear stain (blue). Images were taken at 100x magnification. Scale bar denotes 10 μm.

We then performed dual staining on WNV_KUNV_-infected Vero cells using antibodies specific to flavivirus proteins known to be involved in replication of viral RNA (anti-NS1 antibody 4G4 [18], anti-NS5 antibody 5H1 [34] and anti-NS3 rabbit sera [21]). Dual staining revealed that the antigen recognised by 3G1 and 2G4 co-localised with all three proteins at these perinuclear regions in infected cells consistent with a component of the virus replication complex (Fig. 5).

### Digestion with double-stranded RNA specific nuclease abolishes binding by 3G1 and 2G4

**Fig 6:**
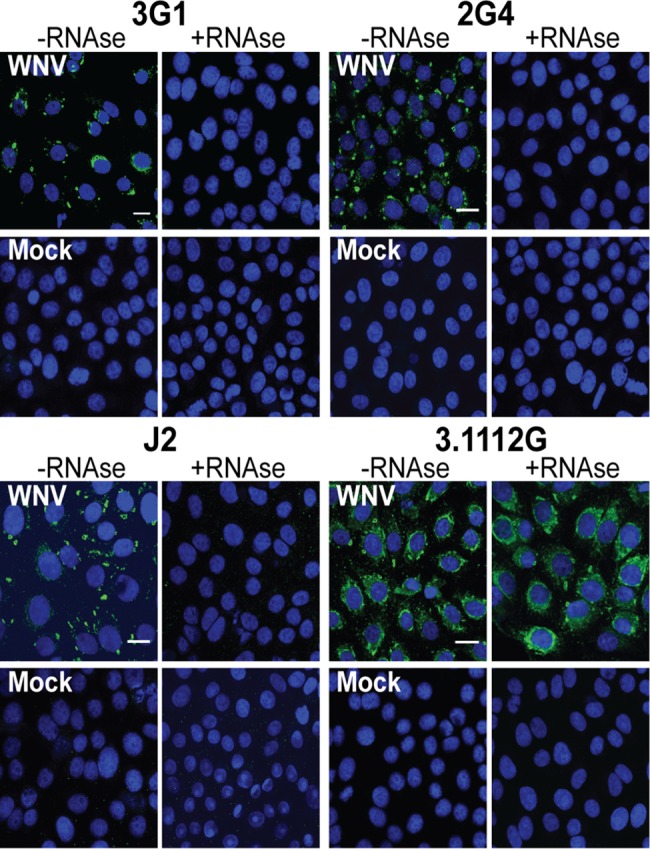
Effect of RNAse III treatment on mAb 3G1 and 2G4 binding. Immunofluorescence assay performed on Vero cell monolayers infected with WNV_KUNV_ at MOI: 10 or mock infected and untreated (-RNAse III) or treated with dsRNA-specific nuclease RNAse III (+RNAse III). Shown are cells stained with mAbs 3G1 or 2G4, anti-dsRNA mAb J2 or WNV_KUNV_ NS1-specific mAb 3.1112G. All antibodies are labelled with goat anti-mouse Alexafluor 488 (green). Nuclei are stained with Hoechst nuclear stain (blue). Images were taken at 40x magnification. Scale bar denotes 10 μm.

Whilst cross-reactivity of these mAbs with divergent viruses suggested that their target antigen was unlikely to be a specific viral protein, the data thus far indicated it was specifically associated with viral infection. The co-localisation of mAb 3G1 and 2G4 staining with proteins of the flavivirus RNA replication complex suggested that the double-stranded replicative intermediate of flaviviral RNA may be a potential candidate for the target antigen. To investigate this we treated acetone-fixed cells with the double-stranded RNA (dsRNA)-specific nuclease RNAse III. This treatment abolished binding for both mAbs 3G1 and 2G4 as well as binding by positive control commercial antibody J2 (Fig. 6) that is specific for dsRNA [20, 35]. Binding of NS1-specific antibody 3.1112G was not affected by this treatment (Fig. 6). The results thus implicated dsRNA as the primary target for 3G1 and 2G4 antibodies

### 3G1 and 2G4 react with double-stranded RNA analogue

**Fig 7:**
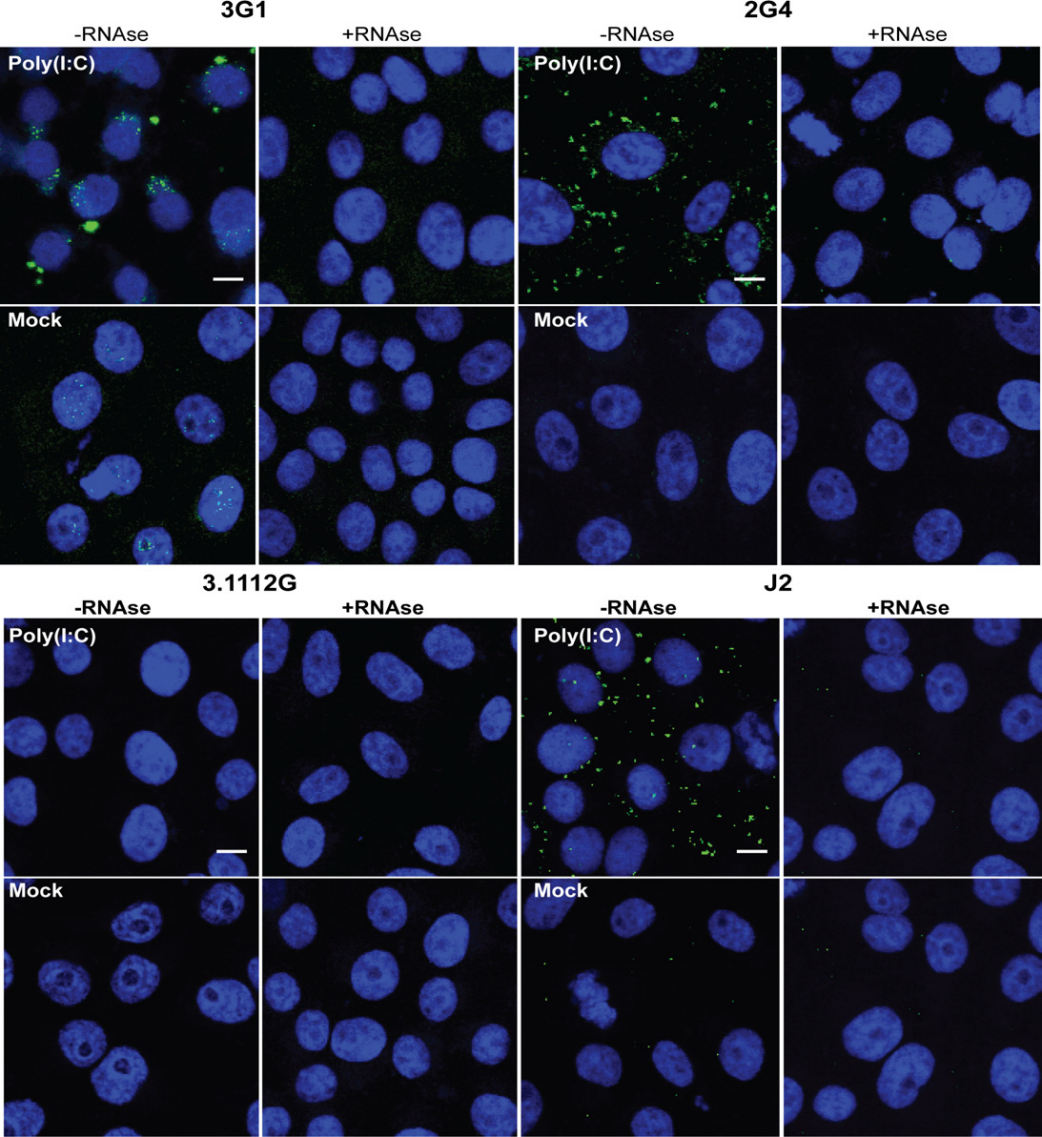
Reactivity of mAbs 3G1 and 2G4 to synthetic dsRNA. A) Immunofluorescence assay performed on Vero cell monolayers transfected with Poly(I:C) or treated with transfection reagent only (mock). Cells were also subjected to pre-treatment with or without RNAse III (+RNAse,-RNAse respectively). Shown are coverslips stained with mAbs 3G1 or 2G4, anti-dsRNA mAb J2 or WNV_KUNV_ NS1-specific mAb 3.1112G. All antibodies are labelled with goat anti-mouse Alexafluor 488 (green). Nuclei are stained with Hoechst nuclear stain (blue). Slides were imaged at 40x magnification. Scale bar denotes 10 μm.

To investigate whether mAbs 3G1 and 2G4 bound dsRNA in cells in a non-sequence specific manner, we tested both antibodies in IFA against acetone-fixed cells which had been transfected with Poly(I:C) [35]. Both 3G1 and 2G4 showed cytoplasmic binding in transfected cells, with only some nuclear staining occurring in mock-transfected cells (Fig. 7). Staining was again abolished when cells were treated with RNAse III.

**Fig 8:**
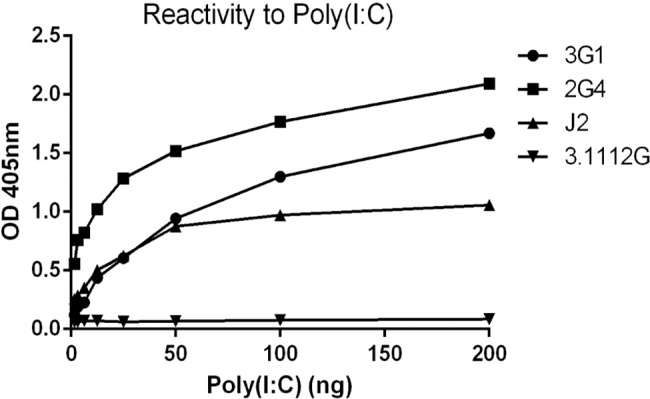
ELISA was performed on 96-well plate coated with 0.25–200 ng/well Poly(I:C). Monoclonal antibodies used were 3G1, 2G4, positive control anti-dsRNA specific mAb J2 and negative isotype (IgM) control anti-NS1 mAb 3.1112G.

To further confirm that the mAbs were binding specifically to Poly(I:C), and not to a Poly(I:C)-induced cellular component, we assessed antibody reactivity in vitro using Poly(I:C)-coated 96-well plates in ELISA [20]. Again, both 3G1 and 2G4 showed strong reactivity to Poly(I:C) in this format (Fig. 8).

### 3G1 and 2G4 detect dsRNA molecules of different sizes and are specific to dsRNA

**Fig 9:**
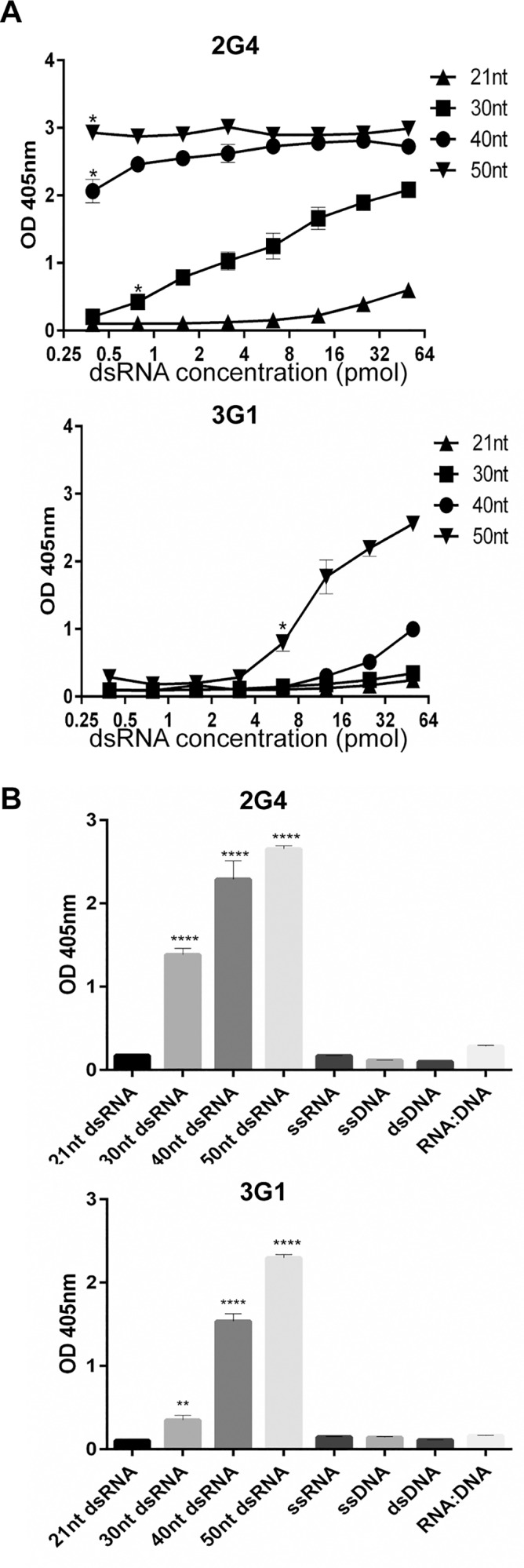
Reactivity of mAbs 3G1 and 2G4 to small nucleic acid oligonucleotides in capture ELISA. Purified mAb 2G4 was coated to 96-well plates at predetermined optimal concentration. MAb 3G1 was concentrated from hybridoma supernatant and coated at a predetermined optimal dilution. Capture of biotinylated oligonucleotides was measured by probing with streptavidin-HRP. A) Reactivity to biotinylated double-stranded RNA molecules (0.25–50 pmol/well) of varying lengths was tested in capture ELISA format. Molecule sizes tested were 21 bp, 30 bp, 40 bp, 50 bp. * denotes concentration at which difference between test samples and negative control (21 bp molecule) becomes significant. Statistical significance was analysed using two-way ANOVA, followed by Dunnett test for multiple comparisons (P<0.0001). B) Reactivity of mAbs to biotinylated nucleic acid molecules was tested in capture ELISA format using predetermined optimal concentrations (5–15 pmol/well). Molecules tested were dsDNA, ssDNA, ssRNA and RNA:DNA hybrid all 50 bp in length. **** denotes significant difference between test samples and the negative control (21 bp dsRNA). Statistical significance was analysed using one-way ANOVA, followed by Dunnett test for multiple comparisons (P<0.0001).

The inability of mAbs 3G1 and 2G4 to bind dsRNA following RNAse III digestion suggested that these mAbs require the dsRNA target to be greater than 18–25 base pairs (bp), since this is the length of molecule produced by RNAse III digestion [36]. In order to further characterise the minimal size of dsRNA recognised by mAbs 3G1 and 2G4, we designed a number of short biotinylated dsRNA molecules (Integrated DNA Technologies). Antibody reactivity was assessed in ELISA by capture of small dsRNA molecules by antibodies coated to plates in solid phase. Purified 2G4 captured both 50 bp and 40 bp molecules strongly with reactivity saturated at 62.5 pmol/ml. Optical density readings showed lower levels of capture for the 30 bp molecule (Fig. 9A). Concentrated antibody 3G1 captured the 50 bp molecule strongly, but appeared less effective for smaller molecules (Fig. 9A). Capture ELISA was also performed on plates coated with a negative isotype control antibody 3.1112G to rule out non-specific binding (S1 Fig).

To confirm the specificity of these mAbs for dsRNA, we then tested against a panel of 50 nt long nucleic acid oligos in capture ELISA (dsDNA, ssDNA, ssRNA, RNA:DNA hybrid). Both 3G1 and 2G4 reacted to the dsRNA molecules but did not show significant reactivity to single-stranded RNA (ssRNA), double-stranded or single-stranded DNA (dsDNA or ssDNA) or a double-stranded RNA:DNA hybrid (RNA:DNA) (Fig. 9B).

Together these data suggested that mAbs 3G1 and 2G4 bound specifically to dsRNA. The different binding profiles in capture ELISA suggest that the mAbs may have different affinities for small dsRNA molecules, with 2G4 able to bind equally well to 40 and 50 bp molecules whilst 3G1 preferentially bound longer (50 bp) molecules.

### 3G1 and 2G4 recognise infection with a wide range of arboviruses

**Fig 10:**
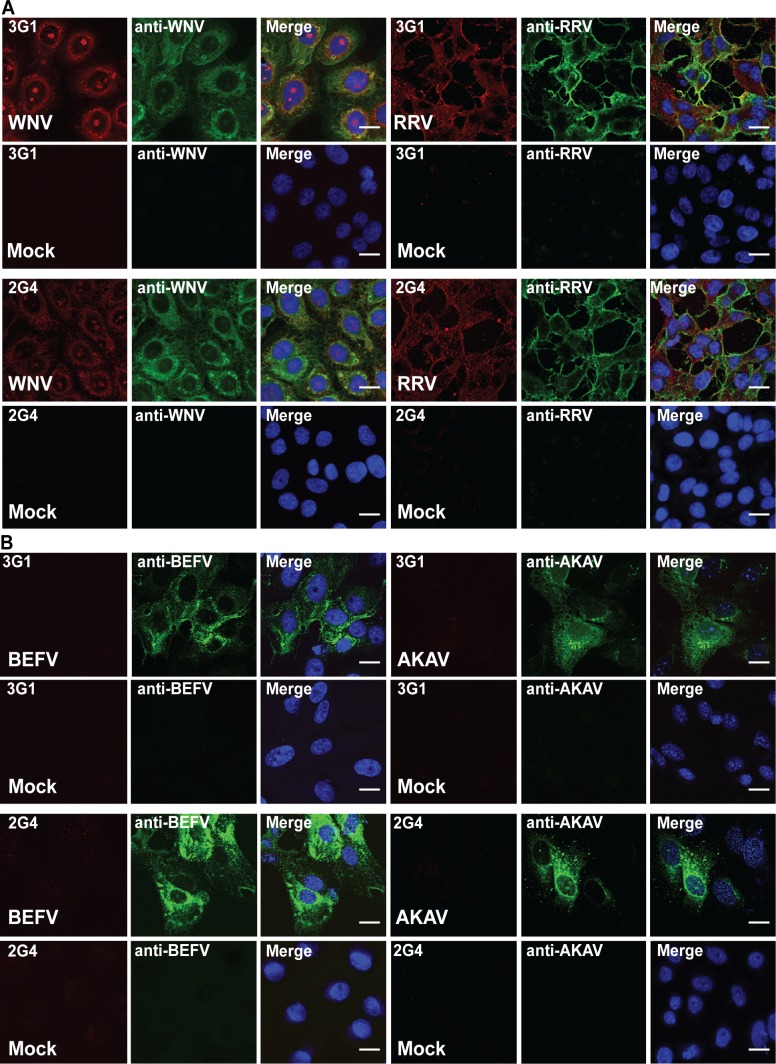
Reactivity of mAbs 3G1 and 2G4 against cells infected with various arboviruses. Immunofluorescence assay was performed on infected and mock-infected Vero cell monolayers. A) Merged images showing dual stained Vero cell monolayers infected with the positive-sense RNA viruses WNV_KUNV_ (*Flaviviridae*; MOI:1, 48 hours post-infection) or (RRV (*Togaviridae*; MOI: 1, 24 hours post-infection) and stained with virus-specific mAbs (green) and 3G1 or 2G4 (red). B) Merged images showing dual stained Vero cell monolayers infected with the negative-sense RNA viruses BEFV (*Rhabdoviridae*; MOI:0.01, 72 hours post-infection) and AKAV (*Bunyaviridae*; MOI: 0.1, 24 hours post-infection) and stained with virus-specific mAbs (green) and 3G1 or 2G4 (red).

To assess the suitability of 3G1 and 2G4 antibodies to detection of a wide range of virus infections, we performed IFA staining of cells infected with representatives of six arboviral families. Dual staining with each mAb 3G1 and 2G4 and antibodies specific for corresponding viral antigens was employed to examine cells infected with the alphavirus Ross River virus (RRV), flavivirus WNV_KUNV,_ and the two negative-sense RNA viruses bovine ephemeral fever virus (BEFV) and Akabane virus (AKAV). The positive-sense RNA viruses RRV and WNV_KUNV_, were both detected in IFA by 3G1 and 2G4 (Fig. 10A) with staining localised in the perinuclear region of infected cells for the flavivirus, while staining in alphavirus-infected cells appeared throughout the cytoplasm consistent with the expected location of replication complexes for the corresponding viruses. Both negative-sense RNA viruses AKAV and BEFV could not be detected with mAbs 3G1 and 2G4 in IFA (Fig. 10B).

**Fig 11:**
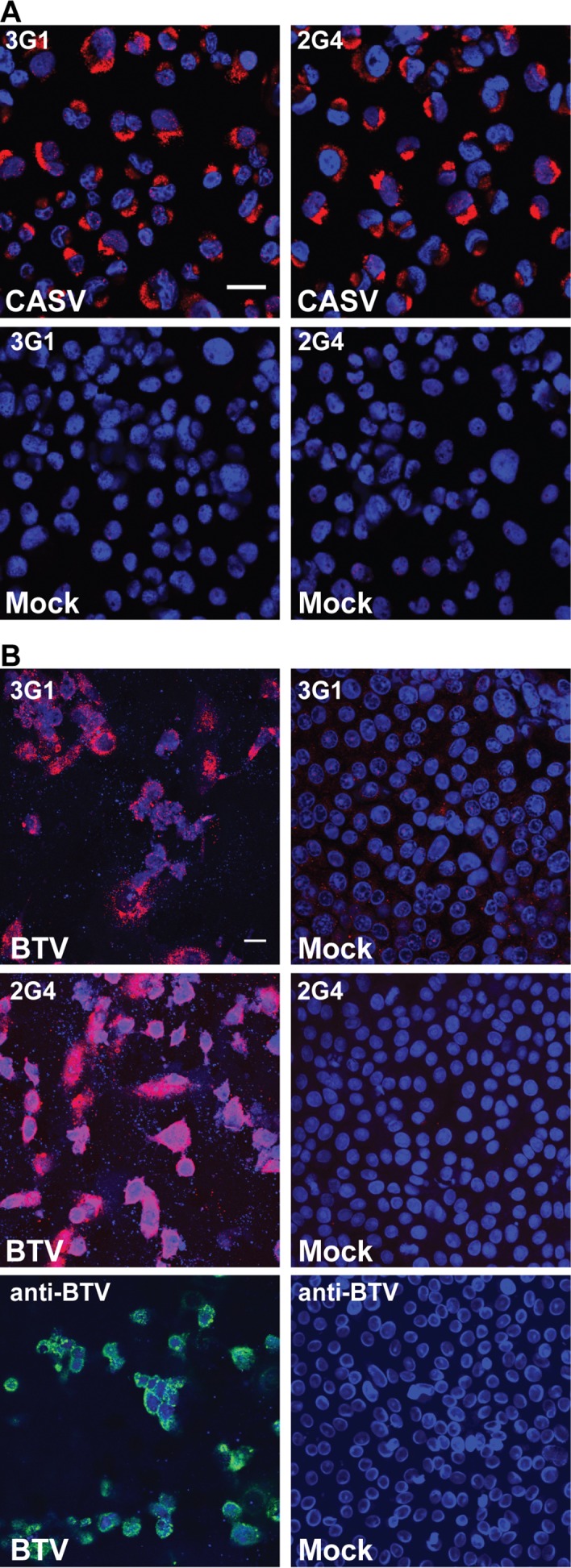
A) C6/36 cell monolayers infected with CASV (*Mesoniviridae*, MOI:1, 72 hours post-infection) or mock infected and stained with mAbs 3G1 or 2G4 (red). B) Vero cell monolayers infected with BTV (*Reoviridae;* MOI:1, 24 hours post-infection) or mock-infected and stained with mAbs 3G1 or 2G4 (red) or BTV-specific mAb (green). Nuclei stained with Hoechst nuclear stain (blue). Images were taken at 40x magnification. Scale bar denotes 10 μm.

Staining in IFA for the, mesonivirus Casuarina virus (CASV), a novel positive-sense RNA virus, displayed a similar perinuclear staining pattern to that observed for flaviviruses (Fig. 11A) [11].

The dsRNA-encoding reovirus bluetongue virus (BTV) was detected in IFA by mAbs 3G1 and 2G4, with staining observed in the perinuclear region of infected cells and coinciding with the locality of the anti-BTV antibody r847 (Fig. 11B). The speckled pattern observed for the nuclear stain and destruction of the monolayer in infected cells was consistent with the induction of apoptosis in mammalian cells by BTV [37, 38].

The results demonstrate that anti-dsRNA antibodies 3G1 and 2G4 can be used to detect infection with a wide range of positive-sense single-stranded and double-stranded RNA viruses but not with negative-sense RNA viruses

Interestingly, in ELISA, mAbs 3G1 and 2G4 showed low reactivity to DENV-infected cells in comparison to WNV_KUNV_-infected cells (Table 1, S2A Fig). While WNV_KUNV_-infected cells showed a marked increase in 3G1 and 2G4 binding in comparison to the mock-infected cells at days 3, 4 and 5 post-infection, there was substantially lower reactivity of the mAbs to cells infected with DENV-2. To further investigate this discrepancy, IFA was performed on cells inoculated with DENV-2 and WNV_KUNV_ under the same conditions as the ELISA, apart from the fixation process which was performed with 100% acetone (*c*.*f*. 20% acetone for ELISA). In IFA, mAbs 3G1 and 2G4 displayed similar reactivity to both WNV_KUNV_ and DENV-2-infected cells, suggesting the presence of similar levels of dsRNA. (S2B Fig). This was further supported by Taqman RT-PCR, whereby high levels of DENV RNA were detected in infected cells at days 3, 4 and 5 (as concluded from the low Ct scores, S2C Fig). Together, these data indicate that the low reactivity of mAbs 3G1 and 2G4 to DENV-infected cells using ELISA is not due to insufficient RNA.

### MAVRIC detects virus infection with similar sensitivity to virus-specific monoclonal antibodies

**Fig 12:**
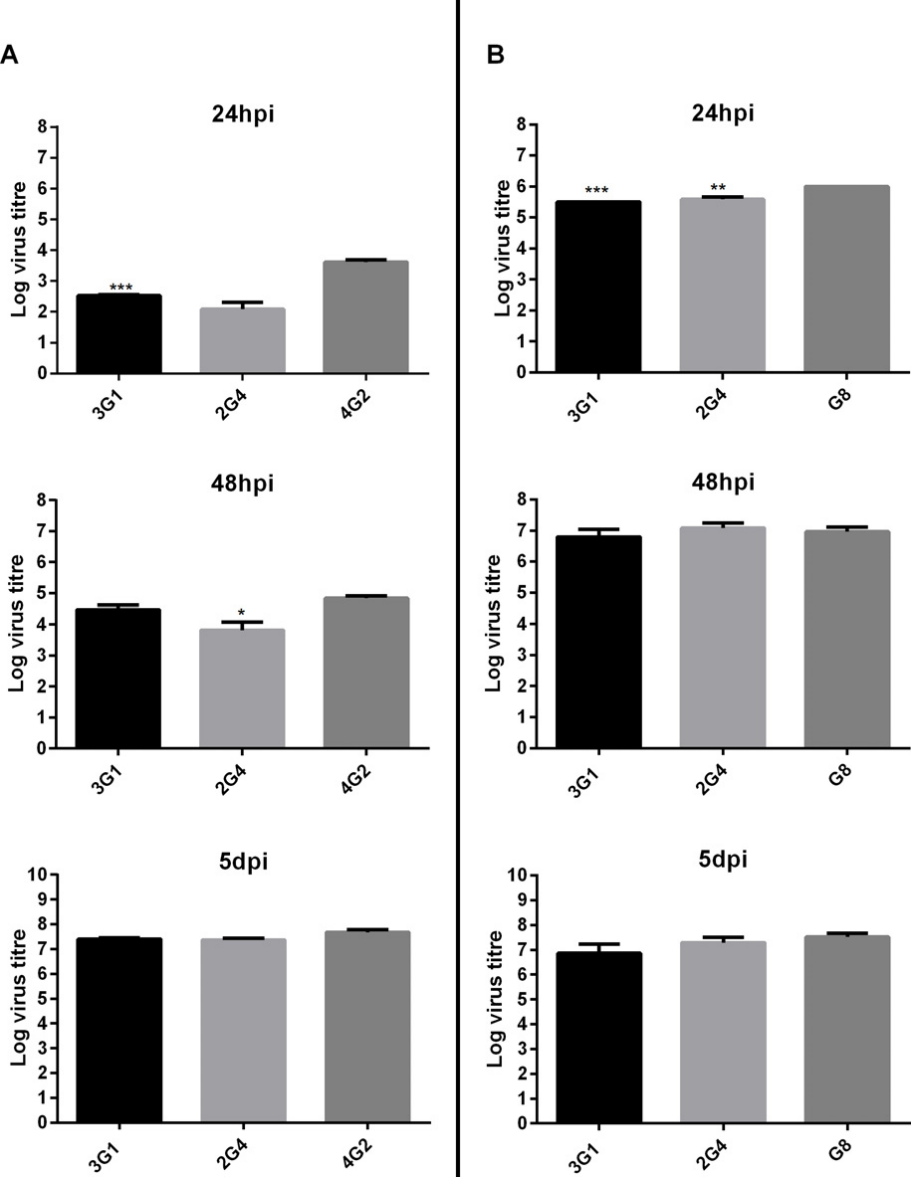
Sensitivity of dsRNA detection in comparison to viral protein of circulating arboviruses at different time points. A) C6/36 cells were infected with WNV_KUNV_ or B) RRV T48 in serial dilutions from 10^-1^ to 10^-12^ and fixed with acetone at 24, or 48 hours post-infection or at 5 days post-infection. Fixed cell ELISA was performed using antibodies 3G1, 2G4 and the virus E protein-specific mAbs 4G2 (flavivirus) or G8 (RRV, alphavirus). Sensitivity is presented as TCID_50_ values for virus detected by each antibody. *** denotes significant difference between level of virus detection by mAbs 3G1 or 2G4 and virus-specific mAb 4G2 or G8. Statistical significance was analysed using one-way ANOVA comparison of means, followed by Turkey test for multiple comparisons (P<0.0005).

To investigate the sensitivity of virus-detection by MAVRIC, in comparison to traditional methods using virus specific mAbs, fixed cell ELISA was performed on C6/36 cells infected with virus at various dilutions after 24 hours, 48 hours and 5 days post-infection. There was no significant difference in the detection of WNV_KUNV_ by mAb 3G1 in comparison to the flavivirus E-protein specific antibody 4G2 at 48 hours and 5 days post-infection. However, at 24 hours post-infection, detection of WNV_KUNV_ infection of at least one log higher was achieved by staining for the E-protein with mAb 4G2 in comparison to using 3G1 to detect dsRNA (Fig. 12A). MAb 2G4 showed similar detection ability to the anti-E mAb only at 5 days post-infection (Fig. 12A). In contrast, both antibodies 3G1 and 2G4 showed similar levels of detection of the alphavirus RRV at 48 hours post-infection and 5days post-infection when compared with the RRV E2 protein-specific antibody G8 (Fig. 12B).

### Monoclonal antibodies 3G1 and 2G4 detect known and unknown viruses in mosquito homogenates

Due to their potential application to virus detection and discovery, mAbs 3G1 and 2G4 were incorporated in an ELISA-based assay for streamlined screening of homogenates of mosquito pools collected around Australia. To assess this system we tested the reactivity of the mAbs against acetone-fixed C6/36 cells which had been inoculated with 40 mosquito pool homogenates which had previously been tested for vertebrate-infecting arboviruses by ELISA with viral antigen-specific mAbs or RT-PCR with genus specific primers. Using these methods, 32 of 40 samples were previously found to be positive for a known arbovirus (Table 2). Using mAbs 3G1 and 2G4 in fixed cell ELISA, 35 out of 40 samples were identified as positive for virus infection in at least one of four inoculated wells (Table 2). RT-PCR was also performed on the supernatant collected from homogenate-inoculated cells prior to fixation using generic primers to detect and confirm virus identification. Of the 35 pools identified as positive by the 3G1 and 2G4 ELISA, four were unable to be confirmed by RT-PCR. Pool 1026 which was previously identified as containing Kokobera virus (KOKV) was negative by 3G1 and 2G4 fixed-cell ELISA as well as RT-PCR suggesting that there was no virus replicating in these inoculated cells. Pools 3740 and 4189, which were negative by fixed-cell ELISA were positive by RT-PCR for WNV_KUNV_ and ALFV respectively, suggesting that these homogenates contained low level of virus below the threshold of detection by fixed-cell ELISA. These pools may become positive in fixed-cell ELISA with further passaging. However, since mAbs 3G1 and 2G4 require the replication of viable virus, it cannot be definitively concluded that viable virus was present in these samples by RT-PCR since detection of residual RNA from the inoculum cannot be ruled out. In addition, of 8 pools which were negative by conventional methods, 6 were identified as positive for virus infection by mAbs 3G1 and 2G4, observation of CPE for 4 of these pools supported the presence of an unknown virus.

**Table 2:** Detection of circulating arboviruses from mosquito homogenates by 3G1 and 2G4.

Pool ID	Year	Original processing results		New processing results		
		Virus Identified*	CPE	3G1/2G4 ELISA^#^	No. of inoculated wells positive (out of 4)	Detection and sequence confirmation by RT-PCR^&^
174	1996	SINV	+	+	4	+
487	1997	SINV	+	+	1	+
494	1997	SINV	+	+	4	+
495	1997	SINV	+	+	4	+
496	1997	SINV	+	+	4	+
979	1998	Negative	-^1^	+	4	-
981	1998	Negative	-^1^	+	4	-
982	1998	Negative	-^1^	+	4	-
1014	1998	KOKV	+	+	4	+
1026	1998	KOKV	+	-	0	-
1049	1998	KOKV	+	+	4	-
1051	1998	KOKV	+	+	4	-
1053	1998	KOKV	+	+	4	+
1197	1999	WNV_KUNV_	+	+	4	+
1268	1999	WNV_KUNV_	+	+	3	+
1274	1999	WNV_KUNV_	+	+	4	+
1284	1999	ALFV	+	+	4	+
1391	1999	WNV_KUNV_	+	+	4	+
1490	1999	WNV_KUNV_	+	+	3	+
1628	1999	WNV_KUNV_	+	+	4	+
1686	1999	ALFV	+	+	4	+
2116	2000	WNV_KUNV_	+	+	4	+
2129	2000	WNV_KUNV_	+	+	4	+
2206	2000	WNV_KUNV_	+	+	4	+
2269	2000	ALFV	+	+	4	+
2279	2000	WNV_KUNV_	+	+	4	+
2472	2000	WNV_KUNV_	+	+	4	+
3342	2000	WNV_KUNV_	+	+	1	+
3404	2000	WNV_KUNV_	+	+	4	+
3618	2000	WNV_KUNV_	+	+	3	+
3740	2000	WNV_KUNV_	+	-	0	+
3777	2000	SINV	+	+	1	-
4009	2000	SINV	+	+	4	-
4079	1999	WNV_KUNV_	+	+	1	+
4189	1999	ALFV	+	-	0	+
4270	2001	Negative	-^1^	+	4	-
4271	2001	Negative	-	+	4	-
4280	2001	Negative	-	-	-	-
4298	2001	Negative	-	+	4	-
4309	2001	Negative	-	-	0	-

Legend: + positive;—negative.

* Viruses previously identified using virus specific monoclonal antibodies in ELISA

# Fixed-cell ELISA using mAbs 3G1 and 2G4 against four wells of C6/36 cells inoculated with mosquito homogenate. Samples were deemed positive if at least one well gave strong positive in ELISA.

^&^RT-PCR using flavivirus- and alphavirus-specific primers was performed on RNA extracted from supernatant harvested from inoculated wells prior to fixing.

^1^CPE was observed in these samples upon subsequent passaging during our analysis.

Negative, no virus was identified by reagents specific for known viruses; SINV, Sindbis virus; WNV_KUNV_, West Nile virus subtype Kunjin; ALFV, Alfuy virus; KOKV, Kokobera virus.

These antibodies have also been instrumental in the detection of unknown viruses from mosquito pools. Using the same fixed-cell ELISA based approach, mosquito homogenates which were determined to be negative for known arboviruses by standard methods, including detection of viral antigen with virus-specific mAbs or viral RNA by RT-PCR, were screened using mAbs 3G1 and 2G4 (Table 3). Based on the reactivity of mAbs 3G1 and 2G4 in fixed-cell ELISA, pools containing a number of viruses were selected for further analysis. Viral RNA sequence was amplified from extracted RNA by RT-PCR using generic virus primers or random primers. Sequence was then obtained by Sanger sequencing or next generation sequencing. Preliminary analysis of these sequences by BLASTX indicated that three of these samples contained previously unidentified viruses (Table 3). A fourth sample (pool no. 3, Table 3) was determined by deep sequencing to contain the reovirus Liao Ning virus (LNV) which was only recently detected in Australia [10].

**Table 3:** Novel virus isolates from mosquito homogenate detected in ELISA by mAbs 3G1 and 2G4.

Pool no.	Sequence length (bp)	Positive ELISA optical density reading *	RT-PCR primer or Next generation sequencing (NGS)	E-value	Identity	Query cover	Closest match	Genbank accession no.
1	863	1.057	NGS	8e-106	68%	95%	Corriparta virus (*Reoviridae*)	AGT51062.1
2	248	1.298	RT-PCR	1e-11	61%	53%	Uncultured virus (RdRP-like protein)	AEA40853.1
3	2785	0.863	NGS	0	98%	94%	Liao Ning Virus (*Reoviridae*)	AAQ83563.1
4	601	0.6	RT-PCR	2e-113	86%	98%	Mediterranean Ochlerotatus flavivirus (*Flaviviridae*)	AEH43703.1

Legend: Preliminary sequence analysis performed using BLASTX

* OD of test sample with mock OD subtracted

## Discussion

We have produced two new monoclonal antibodies which recognise viral dsRNA present in the cytoplasm of cells infected with most positive-sense RNA and double—stranded RNA viruses. We have also demonstrated the potential of these reagents for the rapid detection and discovery of novel viruses from diverse viral families in biological samples.

Double-stranded viral RNA is produced in cells infected with most positive-sense RNA viruses as an intermediate of genomic RNA replication. This process has been well characterised for the flavivirus WNV_KUNV_ which was used as the reference virus in our studies. During flavivirus replication, the genomic RNA is used as a template for synthesis of a negative-sense complementary strand leading to the formation of dsRNA products referred to as the replicative form (RF), from which new positive-sense RNA is generated [39, 40]. The flavivirus non-structural (NS) proteins (e.g. NS3, NS2A and NS5) form a replication complex (RC) with the RF in membranous compartments derived from the endoplasmic reticulum known as vesicle packets (VPs). Within the VPs, the RC and RF is anchored to the membrane via interactions with other membrane-associated non-structural proteins including NS4 and NS1[21]. Using dual staining in IFA we have demonstrated co-localisation of mAb 3G1 and 2G4 staining with several NS proteins involved in the RC (NS1, NS3 and NS5), confirming their specific recognition of viral dsRNA produced during flavivirus replication. Strong, specific staining by 3G1 and 2G4 of cells infected with the (+)ssRNA viruses CASV (*Mesoniviridae*), RRV (*Togaviridae*) and the dsRNA virus BTV (*Reoviridae*), was also consistent with the expected location of viral dsRNA in these cells [7, 41–43].

In contrast, no dsRNA was detected by these mAbs in the cells infected with the negative-sense RNA ((-)ssRNA) viruses BEFV (*Rhabdoviridae*) and AKAV (*Bunyaviridae*). These results are consistent with the findings of others who observed that an anti-dsRNA mAb (J2) did not bind cells infected with the negative-strand RNA viruses influenza A virus (*Orthomyxoviridae*) and LaCrosse virus (*Bunyaviridae*) [35]. This can be explained by the genome replication strategy of these viruses. Both rhabdoviruses and bunyaviruses encapsidate their genomic and anti-genomic RNA by complexing them with viral nucleoprotein during synthesis [44–47]. Presumably this prevents the formation of long stretches of dsRNA by obstructing complementary base pairing of the genomic and anti-genomic RNA. This enables the virus to avoid induction of cellular antiviral responses such as RNAi and interferon signalling, of which dsRNA is a potent activator [48, 49].

The ability of these antibodies to bind native viral dsRNA as well as the synthetic dsRNA analogue Poly(I:C) which contains only two nucleotides (inosine and cytidine), suggests a sequence-independent binding mechanism, similar to that reported for another anti-dsRNA mAb (J2) [20, 50]. Sequence-independent binding is a common mechanism for dsRNA recognition by dsRNA binding proteins (dsRBPs). Current models for non-sequence specific interaction of these proteins with dsRNA suggests that their dsRNA binding domains (dsRBDs) interact with the sugar-phosphate backbone of the RNA molecule at successive minor and major grooves of the double helix [51, 52]. Thus, the duplex form is suggested to be the major determinant for substrate recognition and differentiation from other nucleic acid duplexes [52, 53]. DsRNA molecules take on the A-form duplex in which the minor and major grooves of the helix are of similar widths [52]. In comparison, dsDNA is primarily found in the B-form which is characterised by wider major grooves and narrower minor grooves. Finally, RNA-DNA duplexes are believed to take on an intermediate form in which the minor groove of the helix is of comparable size to the A-form but the major groove is significantly larger [52, 53]. Given these previous insights, the specificity of both 3G1 and 2G4 to dsRNA and not to any other nucleic acid species tested, suggests that they recognise dsRNA via the A-form sugar-phosphate backbone in a manner similar to cellular dsRBPs. The sequence-independent recognition of dsRNA by these mAbs highlights their potential as useful tools for discovery and identification of unknown and novel viruses from biological samples.

While most vertebrate-infecting flaviviruses tested in fixed-cell ELISA were detected using mAbs 3G1 and 2G4, we were only able to demonstrate limited detection of DENV in cell culture by this method (S2A Fig). Interestingly, when DENV-infected cells were tested by IFA performed in parallel, mAbs 3G1 and 2G4 showed reactivity to DENV-infected cells similar to that observed for cells infected with WNV_KUNV_ (S2B Fig). Quantitative PCR analysis of viral RNA present in infected lysates suggested that this discrepancy was not due to lower levels of viral replication in DENV-infected cells, and was unlikely to be due to an issue with sensitivity on the part of the MAVRIC ELISA (S2C Fig). We hypothesise that the lower concentration of acetone used in fixation for fixed-cell ELISA may not be sufficient for exposure of dsRNA to mAbs in DENV-infected cells. This warrants further investigation.

Finally, using a streamlined ELISA-based system we have demonstrated the potential of these mAbs for broad-spectrum surveillance of mosquito populations for infection with known circulating arboviruses. This system has been instrumental in the detection of four novel virus isolates from mosquito homogenates using a streamlined ELISA-based system, including LNV which until recently was not known to be present in Australia [10]. We have demonstrated that these antibodies detect flavivirus and alphavirus infection at 5 days post-infection with similar sensitivity to viral protein-specific antibodies commonly used for arbovirus surveillance. In addition, antibody 3G1 detected similar levels of RRV- and WNV-infection as the corresponding virus-specific mAbs at 48 hours post-infection. In contrast, 2G4 was able to detect RRV-infection to similar levels at 48 hours post-infection but only detected similar levels of WNV at 5 days post-infection. This suggests that mAb 3G1 is more sensitive than 2G4, which may be due to the differing affinities of the two antibodies.

Blind analysis of previously processed field samples using fixed-cell ELISA with mAbs 3G1 and 2G4 demonstrated the high sensitivity of this method as well as the ability to detect viruses which were not detected by conventional means. Of the 32 samples that were positive for known viruses by conventional methods, the fixed-cell ELISA with mAbs 3G1 and 2G4 detected all but 3. One of these samples (pool 1026) was also negative by RT-PCR suggesting that the virus in this sample was no longer viable. Two samples were negative by fixed-cell ELISA using mAbs 3G1 and 2G4, but positive by RT-PCR (pool 3047, WNV_KUNV_; pool 4189, ALFV), suggesting that if viable virus was present in these samples, that it was below the threshold of detection of this assay, leading to a false negative rate of 2/32 (6.25%). However, it must be highlighted that the detection of residual RNA from the inoculum by RT-PCR is possible and thus it was not confirmed whether viable virus was present in these samples. An additional six samples that were not positive by virus-specific ELISA or RT-PCR were positive by the 3G1 and 2G4 fixed-cell ELISA, subsequent analysis of these samples found that four pools caused visible CPE indicating the presence of replicating virus in these samples. Two of these samples which were negative for known viruses (pools 4298 and 4271), were positive by 3G1 and 2G4 ELISA but did not present with overt CPE. This highlights the power of this assay for the detection of novel viruses which may otherwise go unnoticed by conventional methods [6].

The two antibodies described here, now referred to as MAVRIC (monoclonal antibodies against viral RNA intermediates in cells), provide a novel approach to broad spectrum virus surveillance and discovery. We have demonstrated the ability of these mAbs to detect infection with viruses from a diverse range of families both in ELISA and IFA with similar sensitivity to that of fixed-cell ELISA using virus-specific monoclonal antibodies. We have recently used these mAbs in our lab to discover and characterise a number of novel flaviviruses, mesoniviruses and reoviruses (Table 3) (9). This system combines the cost-effective platform of fixed-cell ELISA, with broad-spectrum detection of viruses based on the presence of dsRNA, independent of sequence. Thus, these antibodies provide a valuable tool for cost-effective, high-throughput screening of both known and unknown viruses in biological samples. Although the focus of our study was on the detection of arthropod-borne viruses in mosquito cells, we have also shown that both antibodies are able to recognise viral dsRNA species in Vero and DF-1 cells, suggesting this approach would also be applicable to the detection and discovery of various mammalian and avian viruses.

## Supporting Information

S1 Fig Reactivity of negative isotype control 3.1112G (anti-NS1) to small nucleic acid oligonucleotides in capture ELISA. Purified 3.1112G was coated to 96-well plates at the same concentration as mAb 2G4. Capture of biotinylated oligonucleotides was measured by probing with streptavidin-HRP. A) Reactivity to biotinylated double-stranded RNA molecules (0.25–50 pmol/well) of varying lengths was tested in capture ELISA format. Molecule sizes tested were 21 bp, 30 bp, 40 bp, 50 bp. B) Reactivity of mAbs to biotinylated nucleic acid molecules was tested in capture ELISA format using the same concentrations as for purified mAb 2G4. Molecules tested were dsDNA, ssDNA, ssRNA and RNA:DNA hybrid all 50 nt in length.
(TIF)

S2 Fig Detection of flaviviruses WNV_KUNV_ and DENV-2 using mAbs 3G1 and 2G4. IFA. A) Time kinetics of viral dsRNA in flavivirus infected cells detected by mAbs 3G1 and 2G4. Fixed-cell ELISA was performed on C6/36 cells infected with WNV_KUNV_ at MOI: 0.1 or mock-infected and fixed over 5 days. Antibodies used were mAbs 3G1 and 2G4, flavivirus NS1-specific mAb 4G4 or flavivirus E protein-specific 4G2. B) Immunofluorescence assay performed on C6/36 cells mock-infected or infected with WNV_KUNV_ or DENV-2 at MOI: 0.1 and fixed at 3, 4 and 5 days post-infection. MAbs 3G1 and 2G4 and flavivirus E-protein specific mAb 4G2 were labelled with goat anti-mouse Alexafluor 488 (green). Nuclei are labelled with Hoechst nuclear stain (blue). Slides were imaged at 40x magnification. Scale bar denotes 10μm. C) CT values from Taqman qRT-PCR analysis of WNV_KUNV_ and DENV-2 RNA levels in infected cells at 3, 4 and 5 days post-infection. Reference is the CT value equivalent to 10^3^ infectious unit equivalents.
(TIF)
