# Supplementary figures and images for: Viral RNA Intermediates as Targets for Detection and Discovery of Novel and Emerging Mosquito-Borne Viruses
Source: PLoS Negl Trop Dis. 2015 Mar 23;9(3):e0003629. doi: 10.1371/journal.pntd.0003629 (PMC4370754; doi:10.1371/journal.pntd.0003629)

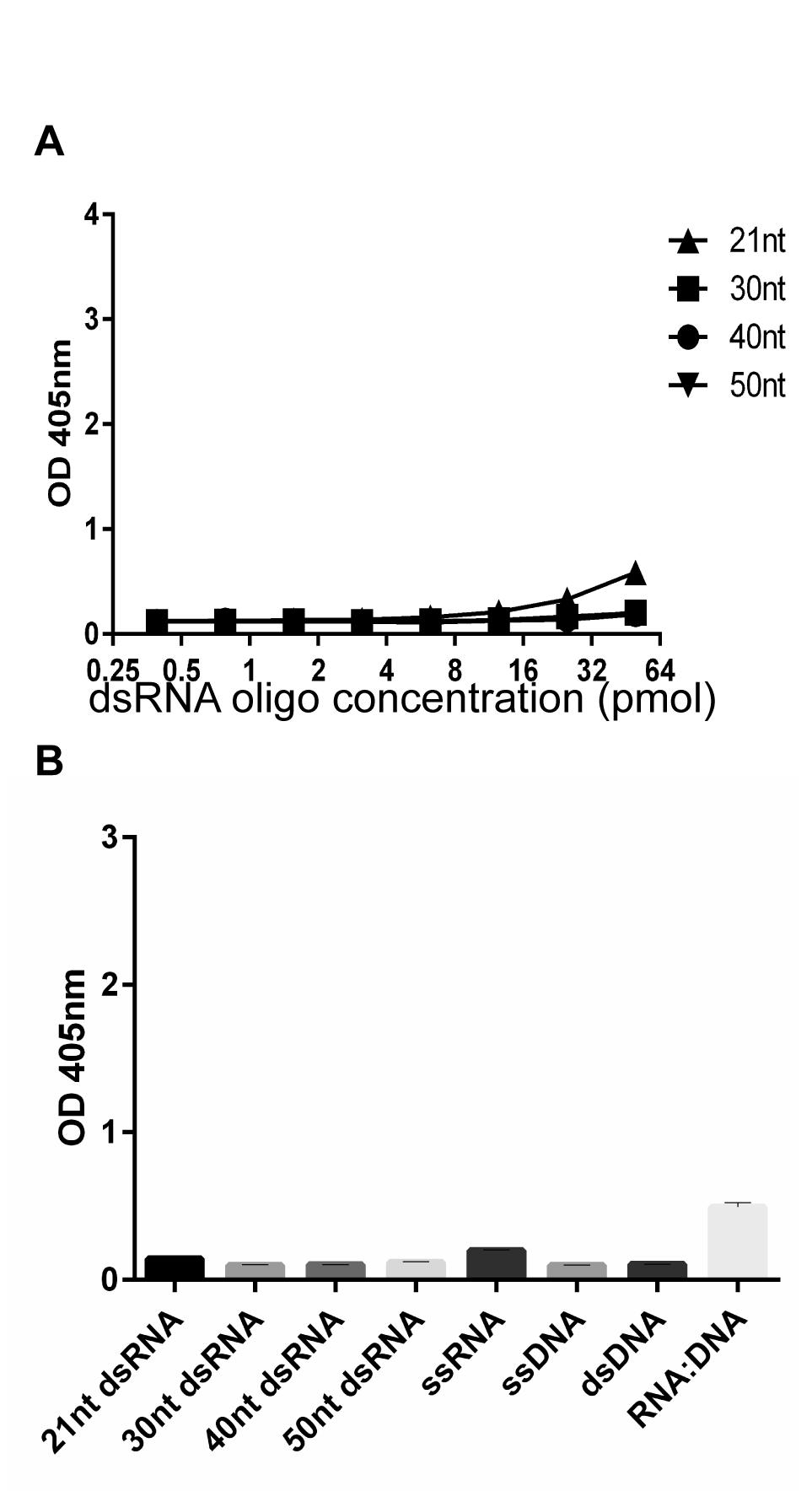

Supplement: S1 Fig — Purified 3.1112G was coated to 96-well plates at the same concentration as mAb 2G4. Capture of biotinylated oligonucleotides was measured by probing with streptavidin-HRP. A) Reactivity to biotinylated double-stranded RNA molecules (0.25–50 pmol/well) of varying lengths was tested in capture ELISA format. Molecule sizes tested were 21 bp, 30 bp, 40 bp, 50 bp. B) Reactivity of mAbs to biotinylated nucleic acid molecules was tested in capture ELISA format using the same concentrations as for purified mAb 2G4. Molecules tested were dsDNA, ssDNA, ssRNA and RNA:DNA hybrid all 50 nt in length. (TIF) [file pntd.0003629.s001.tif]

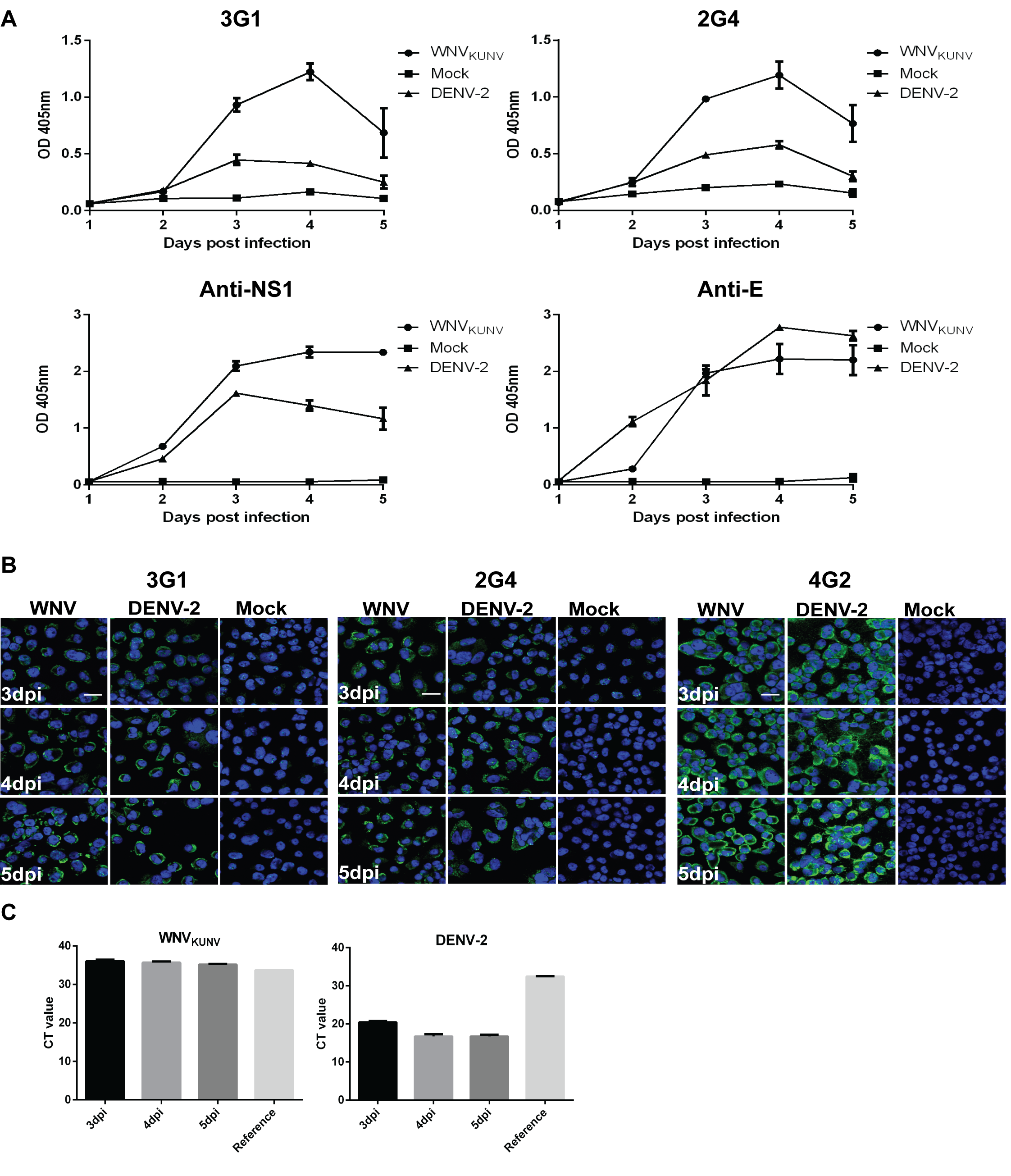

Supplement: S2 Fig — A) Time kinetics of viral dsRNA in flavivirus infected cells detected by mAbs 3G1 and 2G4. Fixed-cell ELISA was performed on C6/36 cells infected with WNVKUNV at MOI: 0.1 or mock-infected and fixed over 5 days. Antibodies used were mAbs 3G1 and 2G4, flavivirus NS1-specific mAb 4G4 or flavivirus E protein-specific 4G2. B) Immunofluorescence assay performed on C6/36 cells mock-infected or infected with WNVKUNV or DENV-2 at MOI: 0.1 and fixed at 3, 4 and 5 days post-infection. MAbs 3G1 and 2G4 and flavivirus E-protein specific mAb 4G2 were labelled with goat anti-mouse Alexafluor 488 (green). Nuclei are labelled with Hoechst nuclear stain (blue). Slides were imaged at 40x magnification. Scale bar denotes 10μm. C) CT values from Taqman qRT-PCR analysis of WNVKUNV and DENV-2 RNA levels in infected cells at 3, 4 and 5 days post-infection. Reference is the CT value equivalent to 103 infectious unit equivalents. (TIF) [file pntd.0003629.s002.tif]
